# Evaluating a dementia risk reduction training program for primary health care educators in Nigeria

**DOI:** 10.1038/s44400-026-00119-2

**Published:** 2026-07-08

**Authors:** Adedoyin. O. Ogunyemi, Adedunni. W. Olusanya, Adedayo E. Ojo, Babatunde. A. Akodu, Abisola T. Omotayo, Victor Valcour, Njideka. U. Okubadejo, Lea.T Grinberg

**Affiliations:** 1 Department of Community Health and Primary Care, College of Medicine, University of Lagos, Lagos, Nigeria; 2 Global Brain Health Institute, University of California, San Francisco, CA USA; 3 Department of Pharmacology, Therapeutics and Toxicology, College of Medicine, University of Lagos, Lagos, Nigeria; 4 Cardiovascular Research Center, University of Abuja, Abuja, Nigeria; 5 Julius Center for Health Sciences and Primary Care, Utrecht University, Utrecht, The Netherlands; 6 University of California, San Francisco, CA USA; 7 Department of Medicine, College of Medicine, University of Lagos, Lagos, Nigeria; 8 Centre for Neurological Sciences Research and Training, College of Medicine, University of Lagos, Lagos, Nigeria; 9 Departments of Laboratory Medicine and Pathology and Neurosciences, Mayo Clinic, Jacksonville, FL USA

**Keywords:** Health care, Medical research

## Abstract

Dementia imposes significant health and economic burdens on low- and middle-income countries, yet nearly 40% of cases are potentially preventable through lifestyle modifications. This study developed and evaluated a dementia risk-reduction training program for 57 primary health care (PHC) health educators in Lagos State, Nigeria, to assess its effectiveness in improving community-level competencies. Using a mixed-methods design, one educator from each local district received training on the WHO mhGAP manual, the ANU-ADRI risk index, and the IDEA cognitive screening tool. Quantitative assessments measured knowledge, skills, and confidence, while focus group discussions explored the intervention’s feasibility and acceptability. Participants were predominantly female (84%), with only 9% having received prior formal dementia training. Post-intervention results showed significant improvements in knowledge regarding symptoms (*p* = 0.023) and caregiver support (*p* = 0.029). Attitudes toward specialist diagnosis (*p* < 0.001) and referral pathways (*p* = 0.018) shifted positively. Furthermore, competency in using risk assessment tools increased significantly (*p* < 0.001). The qualitative findings showed improved confidence, skill application, and perceived community benefits, despite resources and referral challenges. Overall, the training program significantly improved participants’ knowledge, attitudes, confidence, and competency, supporting integration into primary health care systems.

## Introduction

Dementia represents a substantial global public health challenge, with an estimated 152 million individuals projected to be affected by 2050, according to the 2019 Global Burden of Disease estimates^1^. Most cases (68%) occur in low- and middle-income countries (LMICs), where healthcare systems often face resource constraints and limited infrastructure for managing chronic diseases^2^. In Nigeria, an estimated 1.4 million families are projected to be affected by dementia by 2050, a burden likely to worsen in the absence of a comprehensive national preventive strategy^3^. Despite this substantial burden, there is currently no cure for dementia, making risk reduction strategies important for lowering its impact^4^. Studies suggest that nearly 40% of dementia cases may be preventable through lifestyle modifications, such as promoting a healthy diet, managing cardiovascular health, promoting physical activity, and improving cognitive and social engagement^5,6^. Prevention potential may be even greater in LMICs, where the higher burden of modifiable vascular and lifestyle risk factors suggests that population-level interventions could yield greater reductions in dementia risk^7^. However, despite emerging global initiatives, the integration of healthy brain lifestyle promotion and dementia risk screening into routine primary care remains limited in low-resource settings^8^. This highlights the urgent need for community-based interventions that improve dementia risk knowledge and encourage risk-reducing behaviors.

Recognizing the need for initiatives to lower dementia risk, several programs were launched, many of them leveraging the broad reach that virtual communication can provide. However, face-to-face interpersonal approaches have also been suggested as more effective, though they are typically more expensive and have a potentially smaller reach^9^. Health educators, in particular, serve as a crucial interface between communities and Primary healthcare centers (PHCs), providing education about available services and promoting healthy behaviors, expanding the availability of and access to basic health services, particularly in hard-to-reach areas^10,11^. Leveraging this workforce could integrate face-to-face initiatives to reduce dementia at an absorbable cost-benefit^12^.

Studies from PHC centers in Lagos, Nigeria, found that frontline providers had suboptimal awareness and screening capacities for common conditions affecting older adults, including dementia^13,14^. While dementia training for PHC providers has been piloted in Nigeria and other African countries, most initiatives have emphasized case recognition and management^8^. The World Health Organization’s Integrated Care for Older People (ICOPE) framework and emerging brain health initiatives emphasize the integration of dementia risk-reduction strategies into primary care^15,16^. However, there is limited evidence from LMICs on how such strategies can be operationalized, particularly through non-clinical PHC teams. The primary objective of this study was to evaluate the effectiveness of a dementia risk-reduction training program in improving knowledge, skills, and confidence among health educators in Nigeria, while the secondary objective was to assess the feasibility, acceptability, and perceived usefulness of the training for community and primary care application.

## Methods

### Study design and setting

This was a mixed-methods study integrating quantitative and qualitative approaches to assess the effectiveness of a dementia risk-reduction training program. The quantitative component evaluated changes in knowledge, attitudes, and confidence following the training, while the qualitative component explored the participants’ experiences, their capability to carry out the intervention components, and the acceptability of the intervention.by end users. This study was conducted in Lagos State, one of 36 states in southwestern Nigeria (West Africa), with 57 local government districts, and a dense population of over 22 million^17^. The Nigerian health system has three levels of healthcare delivery: primary, secondary, and tertiary. The primary level of care is the entry point of community members into the health system and is comprised of primary healthcare centers administered by the local governments in which the PHC is located^18^.

### Study population and sampling

Participants included adult health educators employed by the Lagos State Government across its 57 districts. Each government district is staffed by approximately 2–3 health educators with similar job descriptions, qualifications, and training requirements, and may be deployed interchangeably across districts. Inclusion criteria required participants to have worked in the current district for at least 6 months. A purposive sampling approach was used to recruit one health educator from each of the 57 districts, selected by the medical officer of health of each district, to ensure full geographic coverage. Similarly, nine health educators were purposively selected from among trained participants for a focus group discussion (FGD), ensuring variation in districts and years of experience.

### Training intervention

#### Training structure, timeline, and resources

The training program consisted of four sessions delivered over a two-month period. The program included two in-person sessions, two online reinforcement sessions, followed by a supervised fieldwork period.

Session 1 (In-person) focused on foundational knowledge including dementia epidemiology, common clinical presentations, types and stages, differential diagnoses, cultural considerations in dementia assessment in the local context, and an overview of dementia risk factors. The session utilized didactic presentations supplemented with case-based discussions drawn from the World Health Organization mental health Gap training manual (Dementia module) for healthcare workers^19^.

Session 2 (In-person) emphasized hands-on practice with the assessment instruments. Health educators practiced administering the Australian National University Alzheimer’s Disease Risk Index (ANU-ADRI), an evidence-based, validated tool aimed at assessing individual exposure to risk factors^20^, and the Intervention for Dementia in Elderly Africans (IDEA) cognitive screening tool^21^, using volunteer participants. Each trainee completed at least two practice assessments consisting of role-play exercises with immediate feedback from trainers.

Session 3 (Online) specifically addressed challenging scenarios such as participants with low literacy, hearing impairment, or initial reluctance to participate. This occurred after the health educators practiced administering the tools on an older adult in the community or PHC setting.

Session 4 (Online) focused on review and calibration, including discussion of ambiguous responses, scoring clarifications, strategies for maintaining participant engagement, and data recording procedures using reference guidelines for administering tools.

#### Pedagogical framework and teaching methods

Our training approach was grounded in adult learning theory, specifically Knowles’ principles of andragogy, which emphasizes that adult learners benefit most from practical, experience-based learning that they can immediately apply to their work^22^. Given that our health educators were experienced community health workers with an average of 10 years of field experience, we designed the training to build upon their existing skills and knowledge. The in-person sessions employed multiple teaching modalities: didactic presentations to establish foundational knowledge, interactive case discussions to promote critical thinking and problem-solving, hands-on practice to develop technical competency with the assessment tools, and role-play exercises to build confidence and address common challenges. The online sessions provided spaced repetition of key concepts, which has been shown to enhance long-term retention^23^, and allowed for individualized clarification of questions. The principal investigator, a public health physician with expertise in brain health and over 12 years of experience in community-based research, an experienced neurologist with 14 years of clinical experience in dementia diagnosis, and a geriatric family physician served as trainers. All trainers had previously completed the training program for trainers using the WHO mhGAP.

#### Supervised fieldwork and quality assurance

Following the four training sessions, health educators completed a structured one-month supervised fieldwork period organized as follows:

Week 1-3: Health educators conducted assessments under direct observation, with supervisors present to provide real-time guidance and feedback. Each health educator completed 20 supervised assessments during this period. Supervisors used a competency checklist covering assessment administration, participant rapport, accurate scoring, and proper data recording.

Week 4: Health educators conducted assessments independently with spot-check supervision, where supervisors randomly observed at least 2 assessments per educator and reviewed all completed data forms daily. Health educators who did not meet competency criteria received additional training and practice until proficiency was demonstrated. Throughout the data collection period, daily team meetings were held to discuss challenging cases, review data quality, and provide ongoing support. The principal investigator reviewed a random 10% sample of completed assessments to ensure continued adherence to protocols.

### Data collection methods and tools

#### Quantitative data

We assessed training effectiveness using a structured questionnaire administered at two time points: immediately before training (baseline) and immediately following the final training session (post-training). The questionnaire (Supplementary file 1) was adapted from the WHO mhGAP dementia module, and included multiple response options per item; to maintain clarity and brevity, only subheadings are presented in the result tables. The knowledge domain assessed health educators’ understanding of dementia fundamentals, formatted as 17 multiple-choice questions and one short answer. The attitude domain measured 11 items on health educators’ beliefs and perceptions about dementia and dementia risk reduction using 5-point Likert-scale items ranging from “strongly disagree” to “strongly agree”. The confidence section measured health educators’ self-reported confidence in assessing risk using 5-point Likert scales ranging from “not strongly confident” to “strongly confident”. Questionnaires were checked for completeness immediately after collection, with participants asked to complete any missing items

#### Qualitative data

We conducted one focus group discussion (FGD) with nine purposively selected health educators six months after the training intervention, ensuring representation across rural and urban districts, gender, and varying levels of professional experience. The selection was based on these participant characteristics to ensure diversity and was not informed by quantitative evaluation outcomes. The timing was deliberately chosen to allow sufficient time for participants to gain practical experience using the tools in community settings, enabling them to reflect on real-world implementation challenges and successes. A semi-structured interview guide was developed to evaluate the participants’ capability of carrying out the components and activities of the intervention, the feasibility of the intervention program, and the acceptability of the intervention by the end users. The FGD took place in a seminar meeting room at the health facility. The session lasted approximately 90 minutes and was conducted in English. At the beginning, the moderator reviewed the purpose of the discussion, obtained written informed consent for participation and audio recording, and established ground rules including confidentiality, respect for diverse opinions, one person speaking at a time, and the option to decline to answer any question. The FGD was audio-recorded using two digital recorders as backup with participants’ written consent. Recordings were transcribed verbatim in English by a professional experienced in transcribing health research interviews. Identifying information was removed during transcription, and participants were assigned codes.

### Measures

Primary Outcomes: Change in knowledge and attitude scores from baseline to post-intervention. Additionally, demonstrated competency in dementia risk assessment and screening. Qualitative measures focused on thematic domains such as knowledge retention and application, impact on professional practice, perceived effectiveness, and barriers to implementation,

### Data analysis

Data analysis was done using descriptive statistics to summarize participants’ characteristics and outcome measures. Numerical data were presented as means and standard deviations. For each of the knowledge items, the number of participants (*n* = 57) providing correct responses was calculated. The degree of change was calculated as the absolute percentage point difference between the proportion of respondents who answered each item correctly at post-test and pre-test (post-test % minus pre-test %) for categorical variables and mean differences for Likert-scale items. Attitude scores were computed as mean scores across all items, with negatively worded items reverse-coded so that higher scores indicated more positive attitudes toward dementia prevention. This measure reflects group-level changes in knowledge and attitude.

Paired t-tests assessed changes in attitude and self-confidence scores, while McNemar’s tests evaluated changes in categorical knowledge and awareness variables. For all analyses, a two-sided *p*-value of <0.05 was considered statistically significant. Missing data were managed using a complete-case analysis approach. Analyses were conducted using Stata version 17.0 (Stata Statistical Software Release 17, 2021; StataCorp LLC, College Station, TX, USA). Thematic analysis followed Braun and Clarke’s six-step framework^24^. Coding was both deductive (applying predetermined codes based on our research questions) and inductive (allowing new codes to emerge from the data), enabling us to systematically address our objectives while capturing unanticipated themes. Dedoose software was used for data management. Reflexivity practices, such as the principal investigator maintaining a journal of observations and reflections, occurred throughout data collection and analysis.

### Ethical considerations

Ethical approval for this study was obtained from the Health Research and Ethics Committee of the College of Medicine, University of Lagos, with approval number CMUL/HREC/10/23/1291. Participants were asked to sign a written informed consent to be included in the study after a thorough explanation of the procedures, risks, and benefits of the study. Confidentiality was ensured by the use of identification codes only, and participants were informed of their right to withdraw from the study at any time without any consequences.

## Results

Of the 57 participants, 48 were female (84%), and 77% had attained a Bachelor’s degree. The mean age was 40.8 ( ± 8.3) years, and 43 (77%) had worked as health educators for less than 10 years, with only five participants (9%) reporting having received formal dementia training within the last two years (Table 1). The knowledge of the impact of dementia improved significantly, with correct answers rising from 70.2% to 89.5% (mean change of 19.3, *p* = 0.019, Table 2). Additionally, knowledge about the common cluster of symptoms in dementia also showed significant improvement in one measure, increasing from 79.0% to 91.3% (mean change of 1.7, p = 0.023). Other areas suggested improvements, but did not meet our threshold for statistical significance. For example, the correct identification of common dementia presentations increased from 80.7% before and 87.7% after (Table 2).

**Table 1:** Summary of participants’ baseline characteristics

Variable	Frequency (*n* = 57)	Percent (%)
**Age (In years)**		
Mean ± SD*	40.8 ± 8.3	-
24–35	16	28.1
36–49	33	57.9
50–56	8	14.0
**Gender**		
Male	9	15.8
Female	48	84.2
**Highest level of education**		
Master’s Degree	13	22.8
Bachelor’s Degree	44	77.2
**Years worked as health educator**		
Mean ± SD*	12.5 ± 7.5	-
<10	43	76.8
≥10	13	23.2
**Had to personally care for an older family member with dementia**		
Yes	14	25.0
No	42	75.0
**Had any formal training specifically related to dementia in the last 2 years**		
Yes	5	8.8
No	52	91.2

^*Treated as a continuous variable.^

**Table 2:** Participants’ knowledge of dementia symptoms, treatment, and caregiver support

	Correctly answered *n* = 57 (%)			
Variable	Pre-test	Post-test	Percentage point difference	*P*-value^†^
Common presentation of dementia	46 (80.7)	50 (87.7)	7.0	0.481
Best description of dementia	51 (89.5)	52 (91.2)	1.7	0.999
Impact of dementia	40 (70.2)	51 (89.5)	19.3	0.019*
Common cluster of symptoms in dementia I	38 (66.7)	46 (80.7)	14.0	0.115
Common cluster of symptoms in dementia II	45 (79.0)	52 (91.3)	12.3	0.023*
Best describes treatment options in dementia	32 (56.1)	44 (77.2)	21.1	0.151
What to do first for a carer of someone with dementia	33 (57.9)	42 (73.7)	15.8	0.307
Best first-line treatment for someone with dementia	37 (64.9)	43 (75.4)	10.5	0.839
Components of psychosocial intervention in dementia	28 (49.1)	30 (52.6)	3.5	0.219
Information to tell a carer of someone with dementia	52 (91.2)	56 (98.2)	7.0	0.029*

^†^McNemar’s chi-square test used to compare pre-test and post-test proportions *Statistically significant

Shifts in attitudes from pre-test to post-test in several areas were noted. The belief that “memory loss is a normal part of aging, so is not worth treating” increased in mean scores from 3.7 to 4.3 (mean difference = 0.6, *p* = 0.027). Similarly, the statement “dementia is best diagnosed by specialist services” saw a substantial increase in agreement, with a mean difference of 0.9 (*p* < 0.001). Other changes include the perception that “it is not worth referring patients to a clinic or hospital as travel is too difficult or expensive,” which increased from 4.1 to 4.6 (mean difference = 0.5, *p* = 0.004). The statement “dementia is a disability rather than a syndrome” also reflected a significant change, with scores rising from 3.4 to 4.5 (mean difference = 1.1, *p* < 0.001). The belief that stigma is attached to having a family member with dementia and the role of the primary health care team in the management and care of dementia did not demonstrate significant changes in Table 3.

**Table 3:** Distribution of participants’ responses to the dementia attitude domain

Attitude Statement	Pre-test Mean	Post-test Mean	Mean Difference	*P*-value^†^
(i) Memory loss is a normal part of aging, so is not worth treating	3.7	4.3	0.6	0.027**
(ii) Dementia is best diagnosed by specialist services	3.5	4.4	0.9	<0.001*
(iii) It is not worth referring patients to a clinic or hospital as travel is too difficult or expensive	4.1	4.6	0.5	0.004*
(iv) Dementia is a disability rather than a syndrome	3.4	4.5	1.1	<0.001*
(v) Providing dementia status for patients and their relatives is usually more helpful than harmful	3.9	4.3	0.4	0.060
(vi) There is stigma attached to having a family member with dementia	3.0	2.5	-0.5	0.052
(vii) The primary health care team has a very limited role to play in the management and care of people with dementia	3.5	3.4	-0.1	0.505
(viii) Family members should take their relative to the hospital to know their relative’s dementia diagnosis as soon as possible	4.0	4.4	0.4	0.018*
(ix) Managing dementia is more frustrating than rewarding.	3.2	3.4	0.2	0.489
(x) The government should play a major role in caring for patients with dementia	4.2	4.4	0.2	0.267
(xi) Much can be done to improve the quality of life of carers of people with dementia and people with dementia	4.4	4.4	0.0	0.659

Items were rated on a 5-point Likert scale (strongly disagree to strongly agree). Higher scores indicate better dementia attitudes. Negatively worded items (i, iii, ix), were reverse-coded.

^†^Paired t-test used to compare pre-test and post-test means *Statistically significant

The mean score for identifying dementia in patients increased from 3 to 4 (mean difference of 1, *p* < 0.0001, Table 4). Participants also reported enhanced skills in administering the ANU-ADRI, with scores rising from 2 to 4 (mean difference of 2, *p* < 0.001). The ability to use the IDEA screening tool improved similarly, from 3 to 4 (mean difference of 1, *p* < 0.001). Additionally, confidence in recommending support services for caregivers increased from 3 to 4 (*p* < 0.001).

**Table 4:** Self-confidence and competency in administering dementia risk tools

Dementia risk tools statement	Pre-test Mean	Post-test Mean	Mean Difference	*P*-value^†^
Have sufficient skills to identify dementia in patients with cognitive impairment symptoms.	3.0	4.0	1.0	<0.001*
Have sufficient skills to administer the Australian National University-Alzheimer’s Disease Risk Index (ANU-ADRI) dementia risk assessment tool	2.0	4.0	2.0	<0.001*
Have sufficient skills to administer the Intervention for Dementia in Elderly Africans (IDEA) screening tool	3.0	4.0	1.0	<0.001*
Can recommend resources of support services available to caregivers and family members of patients with dementia.	3.0	4.0	1.0	<0.001*

^†^Paired t-test used to compare pre-test and post-test means *Statistically significant

In terms of participants’ awareness of lifestyle-based dementia prevention strategies (open-ended), the need for regular physical activity showed the percentage of correct responses increased from 54.4% in the pre-test to 73.7% in the post-test, with a mean difference of 19.3 and a significant *p*-value of 0.041 (Table 5). Eating a balanced diet showed an increase in correct responses (from 47.4% to 59.6%), but this change was not statistically significant (*p* = 0.178). Adequate rest and sleep demonstrated a significant increase from 28.1% to 47.4% (mean difference of 19.3, *p* = 0.019).

**Table 5:** Awareness of lifestyle-based dementia prevention strategies

	Answered correctly *n* = 57 (%)			
Variable	Pre-test	Post-test	Percentage point difference	*P*-value^†^
Regular physical activity	31 (54.4)	42 (73.7)	19.3	0.041*
Eating a balanced diet	27 (47.4)	34 (59.6)	12.2	0.178
Reducing alcohol	24 (42.1)	33 (57.9)	15.8	0.083
Adequate sleep/rest	16 (28.1)	27 (47.4)	19.3	0.019*

^†^McNemar’s chi-square test used to compare pre-test and post-test proportions *Statistically significant

### Qualitative findings

A total of nine health educators (7 females and 2 males) with a mean age of 38 ± 4.7 years participated in the FGD. Four main themes were identified from the interviews as follows^1^: improved knowledge retention and understanding^2^; application of knowledge and skills to community settings^3^; perceived effectiveness and impact on professional practice, and^4^ multilevel barriers to implementation of dementia risk reduction initiatives.

### Theme 1: Training improved knowledge retention and understanding

Before the training, participants described having misconceptions about dementia, many rooted in a culture that conflated cognitive decline with mental illness or spiritual affliction. Health educators with lower educational qualifications described these beliefs most vividly, recalling how they had previously responded to older adults exhibiting behavioral or memory changes with confusion or avoidance rather than clinical assessment. The training helped shift understanding, as participants described recognizing dementia as a distinct progressive neurological condition rather than a sign of madness, which was meaningful for some on a personal level. Six months post-training, all participants demonstrated an accurate understanding of dementia as a neurodegenerative condition, and several described proactively correcting misconceptions among family members and community peers.

“*Someone with dementia, it’s not as if they are mad because they could be aggressive sometimes, but we just need to show them more care and love.”-* P2, Female

Participants with higher educational backgrounds articulated the clinical features of dementia with greater specificity, reflecting the depth of knowledge gained.

“*It is characterized by memory loss, behavioral change, and then, it is not only associated to the aged…it is also a progressive condition that can worsen over time.”-* P4, Female

“….*sometimes it affects their communication, problem solving, some of them don’t even care about themselves anymore*..” – P3, Female

### Theme 2: Application of screening tools in primary care and community settings

Participants described incorporating screening tools into their day-to-day work in ways that were often opportunistic and context-driven rather than systematic. Those in urban primary health care centres, where encounters with older patients were more frequent, reported using the tools most regularly, describing them as an extension of routine consultations. Rural health educators, by contrast, described applying tools primarily during community outreach and home visits, where the pace of interaction and absence of time pressure afforded more opportunity for extended assessment. Participants described the tools as helping them start conversations with families about memory and aging, which they had not previously done. Overall, they showed willingness to use the tools but faced structural and logistical challenges in doing so.

Some participants described applying tools immediately and spontaneously following the training, prompted by recognition of symptoms in people already known to them:

“*Immediately after the [training] exercise, somebody very close to me complained of the mother, an aged woman who used to behave funny… I had to administer one of the assessment tools*.” *–* P7, Male

Others described a near-inevitability of encountering dementia-related symptoms during community work, suggesting the training had sensitized them to a previously unrecognized burden:

“*There’s no way in one month or thereabout, especially when we do our community work, sensitization, and so on, there’s no way you would not see someone that comes with one symptom or two of this dementia*.” *–* P8, Female

*“In the community, if they have anybody with symptoms, we fill the form and refer to the appropriate facility closest to them*.” *–* P1, Female

### Theme 3: Positive impact on professional confidence and routine practice

Across accounts, confidence emerged as a shift from uncertainty about how to respond to dementia presentations to a felt sense of competence and direction. Participants who had prior exposure to older adult care described this transformation as additive: the training gave formal language and structure to intuitions they had previously acted on without a clinical framework. Those without such prior experience described a more foundational change, recounting how they had previously felt unable to respond meaningfully when families raised concerns about aging relatives. The training appeared to change how they positioned themselves in those encounters as an informed resource. This confidence went beyond tool use, as participants felt better able to communicate with families, address concerns, suggest care options, and engage empathetically with affected individuals.

Participants described moments of applying their new knowledge in real situations, often with family or community members outside the training, which reinforced and consolidated what they had learned:

*“I was able to address a particular condition when somebody was having issues with his aged parent, too. So, I addressed it as regards the knowledge gotten from the training that it is a result of memory loss.” –* P1, Female

The shift in communication style was equally prominent, with participants describing a move from directive or dismissive responses toward empathetic engagement that acknowledged the patient’s experience while guiding them toward constructive action:

*“The training taught me how to be empathetic with people… now I can be like ‘ok, ok, this is how [the condition] seems, yes but do you know you could also do it this way?” –* P9, Male

### Theme 4: Multilevel barriers to implementation of dementia risk reduction

While participants were broadly motivated to apply their new skills, implementation was shaped by a set of intersecting barriers that differed in character across settings. Urban health educators described feeling caught between the demands of high patient volumes and the time-intensive nature of dementia screening and counseling, often having to make pragmatic decisions about when and with whom to use the tools. In rural contexts, the barriers were less about time than about communication as participants described the particular difficulty of conducting cognitive assessments with older adults who fatigued quickly, became agitated, or required family member support to complete responses. Across both settings, the referral process was a major problem. While participants could identify potential cases, they felt limited because they knew families would either refuse or could not afford tertiary care. This led to a specific type of professional stress where staff knew the correct steps to take but lacked the resources and systems to actually perform them. Several participants reflected on the gap between what the training had prepared them to do and what the health system enabled them to do, articulating a tension that remained unresolved at the time of the interview.

The challenge of communicating with cognitively impaired or distressed patients was described with particular vividness by rural participants:

*“They get tired easily when you are interacting with them, they are aggressive… when you are asking them a question, you need to come down to their understanding… they could not really provide that full answer except with the support of a family member.”* – P3, Female

The financial barrier to referral was articulated with equal directness, with participants describing the specific moment of tension between recommending care and anticipating the family’s response:

*“The tight schedule we have as health educators… And when you now tell them that they’ll be going to the tertiary hospital where they can get help, they’d take off, because of the financial implication.” –* P7, Male

## Discussion

This study evaluated the effectiveness of a dementia risk-reduction training program for health educators in Nigeria, and the quantitative results demonstrated that the training significantly improved participants’ knowledge, attitudes, and competencies related to dementia risk assessment in primary health care and community settings. These results align with similar studies in Uganda^25^, India^26^, the United Kingdom^27^, and the United States^28^, which show that structured training interventions can improve health workers’ knowledge and competencies in dementia awareness and risk reduction. This study provides context-specific evidence from West Africa, where dementia training initiatives are nascent, and health systems face multiple competing priorities^8^.

In our study, dementia knowledge and competence improved modestly from pre- to post-test, similar to findings among caregivers where increases were observed in both dementia knowledge and sense of competence^29^. Likewise, general practitioners in another study demonstrated significant improvements across all knowledge subscales following a workshop^30^. These findings demonstrate the effectiveness of structured training in improving dementia-related knowledge and skills across diverse groups, although the magnitude of improvement may vary by target audience and intervention design. The statistical significance was concentrated in items directly addressing dementia’s impact (*p* = 0.019), symptom clusters (*p* = 0.023), and carer information (*p* = 0.029) (Table 2). This pattern was reflected in the qualitative data, where participants described a shift that was especially marked among those who had previously equated the condition with madness or insanity. This suggests that the training was most effective in challenging pre-existing misconceptions rather than building entirely new knowledge.

While our study demonstrated improvements across several attitude measures, certain beliefs remained unchanged, including the perception that stigma is attached to having a family member with dementia (*p* = 0.052), and beliefs about the limited role of the primary health care team (*p* = 0.505). Recent findings from the 2024 Alzheimer’s Disease International report indicate persistent stigmatizing attitudes toward people with dementia among the general population, with no measurable improvement since 2019^31^. These findings reflect that deep-rooted stigmatizing beliefs may be more resistant to change through brief educational interventions^32^. The persistence of stigma-related attitudes in our study suggests that addressing misconceptions about dementia may require sustained, multi-level approaches beyond individual training programs^33^. Family stigma, in particular, may be influenced by broader cultural narratives about aging, mental health, and caregiving responsibilities that extend beyond clinical understanding of the condition^34^. Our findings highlight the need for longer-term follow-up studies to assess whether initial attitude improvements translate into sustained behavioral changes. This interpretation is reinforced by FGD participants who described how community members and families resisted referral and disclosure of dementia diagnoses, reflecting the same stigmatizing dynamics that the quantitative attitude items failed to shift. These attitudes suggest they are structurally and culturally embedded rather than insufficient knowledge^35^.

This study observed significant improvements in participants’ self-confidence and competency in using dementia risk tools, with mean scores increasing by 1.0–2.0 points across all four competency items (all *p* < 0.001, Table 4). This finding is in keeping with other studies^36,37^. Similarly, a Vietnam study found that primary care physicians showed sustained improvements in dementia detection skills and self-confidence following brief educational interventions. Our findings suggest that regular exposure to dementia cases through community health activities may help maintain competency levels. From the qualitative data, participants specifically described feeling equipped to administer the ANU-ADRI and IDEA tools (Theme 3), which aligns with the 2.0-point improvement in ANU-ADRI administration skills (Table 4). The integration of these findings suggests that the training’s impact on confidence was driven by skill acquisition rather than general motivation alone.

A relatively high level of baseline knowledge observed among participants may reflect this specific cadre whose roles routinely involve health promotion and community sensitization, and who may therefore have greater prior exposure to health information compared to other PHC workers. However, this also suggests that the sample may not be fully representative of the broader PHC workforce in Nigeria. The IDEA cognitive screening tool has shown validity in community settings in sub-Saharan Africa, including Nigeria and Tanzania^21,38^. Importantly, the improvement observed among health educators suggests that dementia risk reduction can be integrated into existing PHC frameworks without requiring highly specialized personnel. This echoes global calls for task-shifting and task-sharing strategies in addressing non-communicable diseases and dementia care^39^. The FGD participants described incorporating screening tools into routine outreach and community work opportunistically, suggesting that the task-sharing model functioned in informal community health contexts. Urban health educators reported a higher frequency of tool use, consistent with greater patient volume, while rural participants described applying tools primarily during outreach, a pattern that points to the context-specificity of implementation and the need for setting-sensitive deployment strategies^40^.

The training also produced significant improvements in awareness of lifestyle-based dementia prevention strategies, particularly regular physical activity (*p* = 0.041) and adequate sleep (*p* = 0.019) (Table 5). These are modifiable risk factors with established evidence in dementia prevention^41^, and their inclusion in the training curriculum reflects a risk-reduction orientation. Non-significant changes in diet and alcohol reduction awareness suggest these domains may require reinforcement through repeated exposure or community-level messaging strategies. The qualitative accounts of participants using their skills during community sensitization activities align with the potential for lifestyle prevention messages to reach populations not engaged with formal health services.

**Fig. 1:**
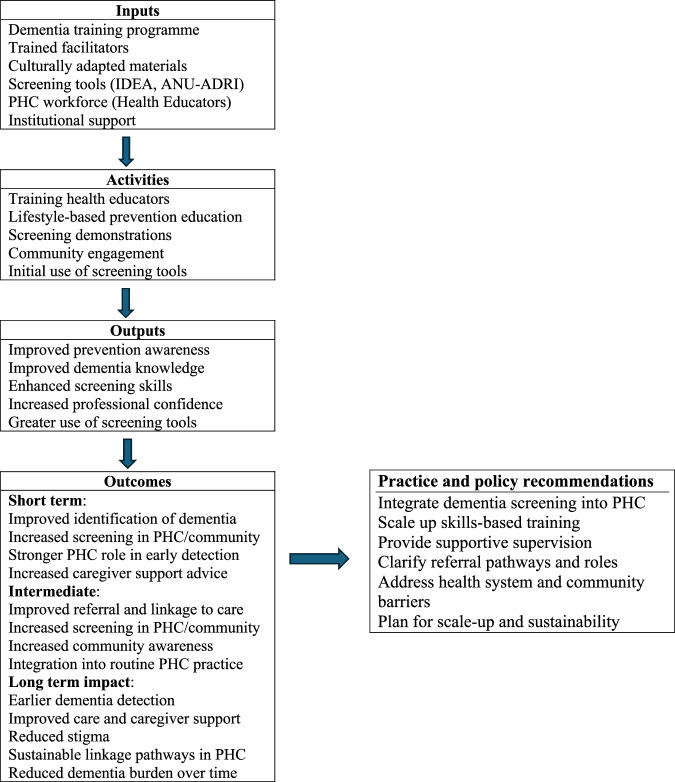
Implementation pathway and recommendations for integrating dementia risk reduction into primary health care.

Despite these encouraging results, challenges such as resource limitations, inadequate referral systems, institutional support gaps, and community stigma directly affect the scalability and sustained impact of training^42^. Addressing these barriers will be essential for long-term integration and impact (Fig. 1). Theme 4 from the FGD described time pressure and high patient volumes as limiting screening frequency among urban health educators, while rural participants identified the financial cost of tertiary referral as a fundamental barrier to the impact of their improved skills. These context-specific barriers suggest that training alone is insufficient to achieve population-level impact, and that investment in referral pathways, follow-up mechanisms, and community stigma reduction must accompany workforce development^14^.

This study demonstrated several strengths, including its mixed-methods design, which enabled the triangulation of quantitative improvements in knowledge, attitudes, and competency with qualitative insights into participants’ experiences and perceived challenges. Focusing on training health educators demonstrated the potential of task-shifting approaches to address the shortage of dementia specialists in Nigeria. However, the study is limited by its relatively small, localized sample of health educators in Lagos State, a relatively resource-rich urban setting; therefore, findings are preliminary and not generalizable to other PHC contexts, particularly rural or lower-resourced settings, but are intended to inform larger future studies. The absence of a control group in this single-group pre–post design means that improvements in knowledge and confidence should be interpreted cautiously. In addition, outcomes were measured only in the short term, meaning that long-term retention of knowledge, sustained practice changes, and community-level impacts remain unknown. Finally, qualitative findings rely on self-reports and may have been influenced by social desirability bias, but this was minimized as the moderator emphasized confidentiality and encouraged experience-based responses^43^.

These findings have important policy and health system implications in Nigeria. The training model aligns with the goals of the National Policy on Ageing for Older Persons in Nigeria^44^, which emphasizes healthy aging, improved quality of life, and strengthening care systems for older adults, including at the community level. By equipping PHC educators with competencies in dementia risk reduction and early recognition, this intervention supports the integration of age-friendly services within PHC and contributes to workforce capacity building. Given that Nigeria currently lacks a comprehensive national dementia strategy despite the growing burden of disease, this training model offers a feasible entry point for embedding dementia prevention and awareness into routine PHC services. It may also inform broader public health strategies focused on risk reduction, early identification, and community engagement, particularly in resource-constrained settings where specialist services are limited.

In conclusion, this study demonstrated that training health educators in dementia risk reduction and screening significantly improved their knowledge, attitudes, and confidence in delivering dementia-related care. The qualitative findings reinforced this, showing that participants felt empowered to engage communities despite contextual challenges. These findings highlight the feasibility and acceptability of integrating dementia risk reduction into primary healthcare in Nigeria. Future programs should scale this training model across broader PHC networks, incorporating supportive supervision and provision of culturally appropriate tools. Further research is needed to test the long-term impact of this intervention on community awareness, early detection, and dementia outcomes, as well as to explore scalability across other low-resource contexts.

## Supplementary information

Supplementary Information

## Data Availability

De-identified data supporting the findings of this study are available from the corresponding author upon reasonable request.

## Code availability

No custom code or mathematical algorithms were developed for this study. All statistical analyses were performed using standard features in Stata version 17.0 (Stata Statistical Software Release 17, 2021; StataCorp LLC, College Station, TX, USA).
