# Supplementary material for: Evaluating a dementia risk reduction training program for primary health care educators in Nigeria
Source: NPJ Dement. 2026 Jul 8;2(1):57. doi: 10.1038/s44400-026-00119-2 (PMC13345960; doi:10.1038/s44400-026-00119-2)
Supplement: Supplementary file 1 — Supplementary Information [file 44400_2026_119_MOESM1_ESM.pdf]

GLOBAL BRAIN HEALTH INSTITUTE

LAGOS STATE GOVERNMENT

ALZHEIMER'S ASSOCIATION

DEMENTIA KNOWLEDGE & RISK ASSESSMENT SURVEY

LGA/LCDA and FACILITY IDENTIFICATION

Name of the LGA/LCDA: ____

Name of affiliated PHC facility: ____

IDENTIFIER: | | | | | | | | | | | |

PARTICIPANT'S PERSONAL CHARACTERISTICS

1	Age (In years)	| | |
2	Gender	Male . . . . 1<br>Female 2
3	What is your highest Level of education?<br><br>Please state your highest health-related degree	Master's degree or above…………...1<br>Bachelor's degree………………………..2<br>Secondary Education or below…….3<br>………………………………………….
4	How long have you worked as a health educator? (In years)	| | |
5	Have you had to personally care for an older family member with dementia?	YES…………………………………………….1<br>NO…………………………………………..2
6	Have you had any formal training specifically related to dementia **in the last 2 years?**	Yes ……………………..........……1<br>No………………………….........…… 2<br>Don't know.....…………………..3

Section A: Knowledge of Dementia

7. Which of the following is a common presentation of dementia? Choose the best answer:

A) Low mood and loss of enjoyment in usual activities.
B) Fixed false beliefs and hearing voices.
C) Excessive hyperactivity and inattention.
D) Decline or problems with memory and orientation.

8. Which of the following statements is a common presentation of dementia? Choose the best answer:

A) Severe forgetfulness and difficulties in carrying out usual work, domestic or social activities.
B) Drowsiness and weakness down one side of the body.
C) Fluctuating mental state characterized by disturbed attention that develops over a short period of time.
D) Low mood in the context of major loss or bereavement.

9. Which of the following is the best description of dementia? Choose only one answer:

A) Dementia is a communicable disease of the brain that can be contagious.
B) Dementia is most common in those aged 40–50 years old, and rare after this age.
C) Dementia is a chronic and progressive syndrome due to changes in the brain.
D) Dementia is rarely noticed by anyone other than the person who has it.

10. Which of the following statements is the best description of dementia? Choose only one answer:

A) Dementia can have a large impact on the person, their carer, family and society at large.
B) Dementia can be cured through pharmacological interventions.
C) Dementia does not interfere with activities of daily living, such as washing, dressing, eating, personal hygiene and toilet activities.
D) Dementia is a normal part of ageing.

11. Which of the following is a common cluster of symptoms in dementia? Choose best answer:

A) Minimally responsive, slow respiratory rate and pinpoint pupils.
B) Problems with orientation, mood and emotional control.
C) Failure to thrive, poor motor tone, delay in reading and writing.
D) Elevated mood, decreased need for sleep, increased activity.

12. Which of the following statements is a common cluster of symptoms in dementia? Choose the best answer:

A) Excessive over-activity and inattention.
B) Excessive crying, clinging to a carer and extreme shyness.
C) Abrupt onset and disturbed level of consciousness.
D) Decline of memory with mood or behavioural problems.

13. Which of the following statements best describes treatment options in dementia? Choose only one answer:

A) All people with dementia should have access to pharmacological interventions, regardless of specialist availability.
B) Pharmacological interventions, if started early enough, can cure dementia.
C) With early recognition and support, the lives of people with dementia and their carers can be significantly improved.
D) Psychosocial interventions for dementia should only be provided by a specialist, due to their complexity.

14. Which of the following might you do first for a carer of someone with dementia? Choose the best answer:

A) Provide them with antipsychotic medication to administer to the person if their behaviour is unmanageable.
B) Provide them with details of specialists, to see if the person can be started on medication.
C) Assess their needs, including whether they are coping or becoming depressed.
D) Refer them to a social worker who can assess whether they are experiencing financial hardship.

15. Which of the following is the best first-line treatment for someone with dementia? Choose the best answer:

A) Pharmacological interventions.
B) Psychosocial interventions.
C) Antipsychotic medication.
D) Referring to a specialist.

16. Which of the following are components of psychosocial intervention in dementia? Choose the best answer:

A) Interpersonal therapy in combination with cognitive behavioural therapy.
B) Promoting independence and support for the person with dementia, including ways to improve cognitive functioning.
C) Cholinesterase inhibitors, in combination with antipsychotics if there are behavioural and/or psychological symptoms.
D) Reducing physical activity, changing their usual routine and leaving things exactly as they are in the house.

17. Which of the following might you tell a carer of someone with dementia? Choose the best answer:

A) The person with dementia will only get worse so you should not bother trying to help them.
B) A lot can be done to make the person with dementia more comfortable and to make providing support less stressful.
C) Taking the person to new and unfamiliar places can help stimulate their memory.
D) The person with dementia should avoid physical and recreational activities to help preserve their health.

Section B: Attitude towards Dementia (tick one)

	**Statement**	**Strongly Disagree**	**Disagree**	**Neither**	**Agree**	**Strongly Agree**
18	Memory loss is a normal part of aging, so is not worth treating					
19	Dementia is best diagnosed by specialist services					
20	It is not worth referring patients to a clinic or hospital as travel is too difficult or expensive					
21	Dementia is a disability rather than a syndrome					
22	Providing dementia status for patients and their relatives is usually more helpful than harmful					
23	There is stigma attached to having a family member with dementia					
24	The primary health care team has a very limited role to play in the management and care of people with dementia					
25	Family members should take their relative to the hospital to know their relative's dementia diagnosis as soon as possible					
26	Managing dementia is more frustrating than rewarding.					
27	The government should play a major role in caring for patients with dementia					
28	Much can be done to improve the quality of life of carers of people with dementia and people with dementia					

Section C: Self-confidence and competency in administering dementia risk tools (tick one)

	Statement	Not strongly confident	Not confident	Neutral	Confident	Strongly confident
29	I have sufficient skills to identify dementia in patients with cognitive impairment symptoms.					
30	I have sufficient skills to administer the Australian National University-Alzheimer's Disease Risk Index (ANU-ADRI) dementia risk assessment tool					
31	I have sufficient skills to administer the Intervention for Dementia in Elderly Africans (IDEA) screening tool					
32	I can recommend resources of support services available to caregivers and family members of patients with dementia.					

Section D: Knowledge of Dementia Risk reduction

33. Mention five lifestyle activities/health-related actions that can reduce dementia risk

a. ______________________________

____

b. ______________________________

____

c. ______________________________

____

d. ______________________________

____

e. ______________________________

____
